# Rocaglamide promotes infiltration and differentiation of T cells and coordinates with PD-1 inhibitor to overcome checkpoint resistance in multiple tumor models

**DOI:** 10.1007/s00262-024-03706-5

**Published:** 2024-06-04

**Authors:** Jiaojiao Luo, Wanyi Ng, Yangli Liu, Lixin Wang, Chenyuan Gong, Yufu Zhou, Cheng Fang, Shiguo Zhu, Chao Yao

**Affiliations:** Present Address: Department of Immunology and Pathogenic Biology, School of Integrative Medicine, Shanghai University of Traditional Chinese Medicine, Shanghai, 201203 China

**Keywords:** Anti-PD-1 antibody, Immune checkpoint blockade, Th17 cells, Rocaglamide

## Abstract

Tumor-infiltrating lymphocyte (TIL) deficiency is the most conspicuous obstacle to limit the cancer immunotherapy. Immune checkpoint inhibitors (ICIs), such as anti-PD-1 antibody, have achieved great success in clinical practice. However, due to the limitation of response rates of ICIs, some patients fail to benefit from monotherapy. Thus, novel combination therapy that could improve the response rates emerges as new strategies for cancer treatment. Here, we reported that the natural product rocaglamide (RocA) increased tumor-infiltrating T cells and promoted Th17 differentiation of CD4^+^ TILs. Despite RocA monotherapy upregulated PD-1 expression of TILs, which was considered as the consequence of T cell activation, combining RocA with anti-PD-1 antibody significantly downregulated the expression of PD-1 and promoted proliferation of TILs. Taken together, these findings demonstrated that RocA could fuel the T cell anti-tumor immunity and revealed the remarkable potential of RocA as a therapeutic candidate when combining with the ICIs.

**Supplementary Information:**

The online version contains supplementary material available at 10.1007/s00262-024-03706-5.

## 1. Materials and methods

### 1.1. Reagents

RocA (BBP00609) was purchased from BioBioPha Co., Ltd. (Yunnan, China). PE anti-mouse CD3(100205), FITC anti-mouse CD4(100405), PerCP/Cyanine5.5 anti-mouse CD8(100733), APC anti-mouse NKp46(137608), purified anti-mouse NK1.1 (PK136) (108702) were obtained from BioLegend Inc. Anti-mouse PD-1(BE0146-RMP1-14) and rat IgG2a(BE0089-2A3) were supplied by BioXcell. Anti-mouse CD45.2(560694), FITC anti-mouse CD3(553061), BV605 anti-mouse CD4(563151), BV510 anti-mouse CD8(563068), BV650 anti-mouse NKp46 (740627), BV421 anti-mouse PD-1 (562584), PE anti-mouse CD107a (558661), PE/Cy7 anti-mouse IFN-γ(557649), PE anti-mouse RORγt(562607), BV421 mouse anti-T-bet(563318), BB700 mouse anti-GATA3(566642), PE-CF594 anti-mouse IL-4(562450), AF647 mouse anti-Ki-67(558615), BV650 anti-mouse IL-17A(564170) were provided by BD Biosciences. APC mouse anti-FOXP3(17-5773-82) was purchased from Thermo Fisher Scientific. PMA(Abs9107), ionomycin calcium salt (Abs9108) were supplied by Absin. Collagenase I(40507ES60) and DNase I(10607ES15) were obtained from Yeasen.

### 1.2. Cell culture

Mouse Lewis lung cancer (LLC) cells, mouse B16F10 melanoma cells and mouse CT26 colon cancer cells were supplied by the Cell Bank of Shanghai Institutes for Biological Sciences, Chinese Academy of Sciences. LLC cells and B16F10 cells were incubated in high-glucose Dulbecco’s modified Eagle’s medium (DMEM) (HyClone, SH30022.01B), and CT26 cells were incubated in RPMI-1640 medium(HyClone, SH30809.01). Both media were supplemented with 10% fetal bovine serum (FBS, Gibco) and 1% penicillin-streptomycin (Yeasen, 60162ES76).

### 1.3. Subcutaneous tumor models

Male C57BL/6 or BALB/c mice were purchased from Vital River Laboratory Animal Technology Co. (Beijing, China), and maintained under a specific pathogen-free (SPF) environment. For subcutaneous tumor models, 1.5 × 10^6^ LLC cells, 2 × 10^5^ B16F10 cells or 5 × 10^5^ CT26 cells were suspended in 100 μL serum-free medium and subcutaneously injected into the right flank of 6-week-old C57BL/6 or BALB/c mice on day 0 and then 1.0 mg/kg of RocA was administered via i.p. injection every 2 days from day 3, anti-PD-1 antibody was administered via i.p. injection every 3 days from day 9. For NK cell depletion, mice were administered with 100 μg of PK136 antibody per mouse via i.p. injection on day 0, 3, 7 10 and 13. The tumor length and width were measured every 2 days using a caliper and the tumor volume was calculated using the following formula: *V* = (*π*/8)*a* × *b*^2^, where *V* = tumor volume, *a* = maximum tumor diameter and *b* = minimum tumor diameter. All animal procedures, including tumor transplantation, tumor volume monitoring and euthanasia, were approved by the Institutional Animal Care and Use Committee at Shanghai University of Traditional Chinese Medicine.

### 1.4. Flow cytometry analysis

Cells were exposed to the appropriate fluorescence-conjugated antibodies at 4 °C for 30 min in the dark, whereas control cells were incubated with the corresponding IgG Fc antibodies under the same conditions, then washed and resuspended in PBS containing 1% FBS. The data were obtained by a BD Accuri C6(BD Biosciences) and analyzed using Flowjo software.

### 1.5. RNA sequencing

1.5 × 10^6^ LLC cells per mouse were subcutaneously inoculated on the upper back of C57BL/6 mice and 1.0 mg/kg of RocA was administered via i.p. injection every 2 days. Mice were sacrificed on day 21, tumors were isolated and analyzed by RNA sequencing, which was performed by Shanghai Biotechnology Corporation. Genes with fold change ≥ 2 and *P* < 0.05 were identified as differentially expressed genes (DEGs).

### 1.6. CD4 depletion in vivo

1.5 × 10^6^ LLC cells were subcutaneously injected into the right flank of C57BL/6 mice on day 0. Tumor-bearing mice were treated with 1.0 mg/kg of RocA via i.p. injection every 2 days from day 3. For CD4^+^ T cell depletion, purified anti-mouse CD4 antibody was intraperitoneally injected with a dose of 50 μg per mouse at 24 h before LLC cell injection and every 5 days until the end of experiments, IgG antibody was used as control for anti-CD4 antibody.

### 1.7. In vitro differentiation analysis of CD4^+^ T cells

Splenic lymphocytes were isolate from healthy C57BL/6 mice and were then cultured in vitro. The cultured lymphocytes were treated by 20 ng/mL of IL-6, 1 ng/mL of TGF-β and 2 μg/mL of anti-CD3 beads and 1 μg/mL of anti-CD28 beads. Intracellular staining of transcription factors and cytokines were conducted to analyze the CD4^+^ T cell subpopulation after 7-day culturing.

### 1.8. Statistical analysis

All data were analyzed using GraphPad Prism software 8.3 (GraphPad, San Diego, CA, USA) and expressed as mean ± standard deviation (S.D.). A two-tailed unpaired *t*-test or one-way analysis of variance (ANOVA) was applied to determine statistical significance (*P* < 0.05).

## Introduction

Cancer immunotherapy has become a prominent strategy in recent years, such as immune checkpoint blockade (ICB) therapy. Programmed cell death protein 1(PD-1), an immune checkpoint molecule expressed on T cells as well as other cells including but not limited to B cells, natural killer (NK) cells and myeloid cells, has received much attention in the past decade [1]. The interaction of PD-1 with its ligands, programmed death-ligand 1 (PD-L1) and programmed death-ligand 2 (PD-L2), can deliver an inhibitory signal and limit effector T cell responses to protect tissues from immune-mediated damage [2], whereas tumor cells take advantage of this signal as a mechanism for immune escape[3]. The high expression of PD-1 is considered to be a marker of functional T cells [4] or exhausted T cells [5], and T cell exhaustion can be reversed by blocking PD-1 signaling [6].

The ICB therapy that bases on blocking the PD-1/PD-L1 axis has demonstrated prominent antitumor activity in multiple cancer types [7–9]. However, the durable remission rates still remain low due to immune resistance, a majority of patients fail to benefit from the therapy as a single agent, thereby novel combination treatments that can overcome the resistance and increase the response rates have attracted extensive attention [10–12]. In patients with solid tumors, responders of ICB therapy exhibit an immune-hot phenotype, characterized by T lymphocyte infiltration, whereas nonresponders may exhibit an immune-cold phenotype, characterized by the absence or exclusion of T cells in the tumor parenchyma [13]. Therefore, promoting T cell infiltration to turn nonresponsive cold tumors into responsive hot ones may become a breakthrough in enhancing ICB therapeutic efficacy.

T cells play a central role in immune-hot tumors, where CD4^+^ T cell release various cytokines that recruit and regulate the activity of other immune cells [14]. Naïve CD4^+^ T cells, under diverse stimulation, differentiate into regulatory T cells (Tregs) and distinct T helper (Th) cell subsets, such as follicular helper T (Tfh), Th1, Th2, Th17, Th9 and Th22 cells. The production of signature cytokines defines Th cell subsets and functional capacities [15]. For instance, Th1 cells are induced by IL-12 [16], Th2 cells are induced by IL-4 [17], while the differentiation of Th17 cells is promoted by IL-1β, IL-6, IL-21, IL-23 and TGF-β [18]. The mature Th cells acquire well-defined functions to combat specific pathogens but are also equipped with plasticity in response to changing microenvironment [19].

As professional cytokine-producing cells, Th cells are an essential and complex component of the immune system. Briefly, Th1 cells can activate macrophages through IFN-γ production [20], Th2 cells have an excellent performance for orchestrating immune responses against extracellular parasites and are involved in the induction of asthma and other allergic diseases by producing IL-4, IL-5 and IL-13 [15]. Th17 cells, capable of producing IL-17, IL-21 and IL-22, mediate immune responses to extracellular bacteria and fungi and are also responsible for different forms of autoimmunity [21, 22]. However, there is a lack of consensus on the role of Th17 cells in cancer progression, due to the plasticity of this subtype, enabling Th17 cell transdifferentiate into other types including Th1, Th2, Treg and Tfh cells in distinct microenvironment [23].

Rocaglamide (RocA), a compound originally isolated from Aglaia elliptifolia, has exhibited significant anti-cancer properties [24]. In our previous studies, we have found that RocA boosts NK cell-based immunotherapy by promoting the tumor infiltration of NK cells via triggering cGAS-STING signaling pathway [25] and targeting autophagy initial gene ULK1 to enhance NK cell-mediated killing of cancer cells [26]. In this study, we observed that RocA facilitated the infiltration and differentiation of T cells, thereby transforming immune-cold tumor into hot ones. When combined with anti-PD-1 antibody, RocA promoted functional activation of CD4^+^ TILs and anti-PD-1 antibody downregulated the PD-1 expression that prevented T cell dysfunction. Furthermore, the depletion of CD4^+^ T cells partially abolished the antitumor activity of RocA in vivo. These findings demonstrated that RocA could promote T cell anti-tumor immunity and had a potential capability in enhancing the response rate of ICB therapy.

## Results

### RocA promotes the intra-tumoral infiltration of CD4^+^ and CD8^+^ T cells

**Fig. 1:**
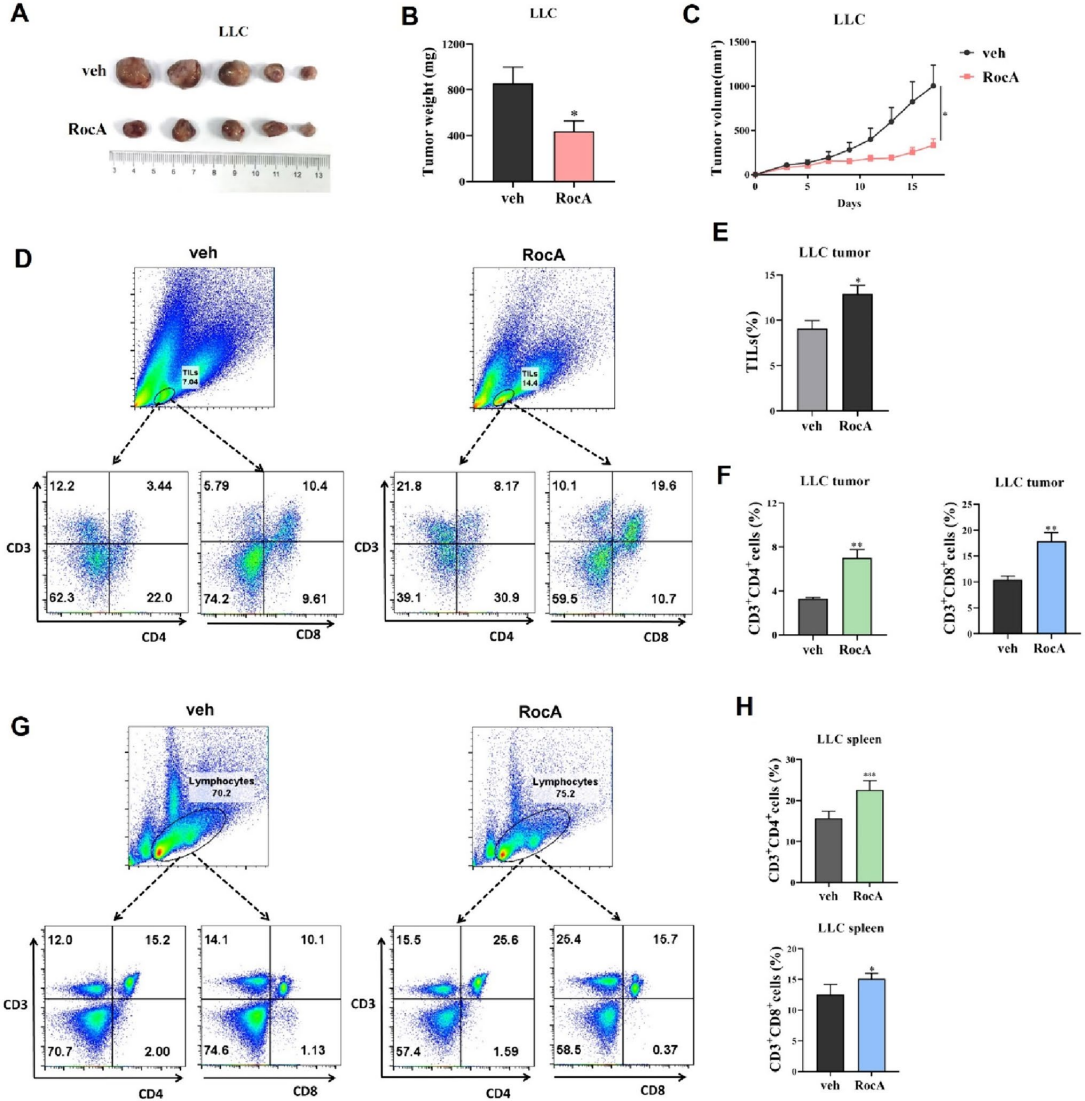
RocA promotes the tumor infiltration of T cells. **A–C** A total of 1.5 × 10^6^ of LLC cells per mouse were subcutaneously inoculated on the upper back in C57BL/6 mice on day 0, and then 1.0 mg/kg of RocA was administered via i.p. injection every 2 days from day 3. Tumor size was measured every 2 days, mice were sacrificed on day 17 and tumors were excised, photographed **A** and weighed **B**. Tumor volume was calculated and tumor growth was plotted **C**. **D**–**F** The tumor tissues were collected on day 17 and TILs (CD3^+^CD4^+^/CD3^+^CD8^+^) were determined through flow cytometry. **G**, **H** The spleens of tumor-bearing mice were collected on day 17 and splenic T cells (CD3^+^CD4^+^/CD3^+^CD8^+^) were determined through flow cytometry. *p < 0.05; **p < 0.01

In the previous studies, we have found that RocA could enhance antitumor activity of NK cell-based immunotherapy. In addition to NK cells, T cells play a critical role in cancer immunotherapy. The previous findings [25, 26] lead us to investigate whether RocA could increase the tumor infiltration of T cells. In this study, we found that RocA suppressed the growth of mouse Lewis lung cancer (LLC) cells in vivo (Fig. 1A), the tumor weight of mice treated with RocA were significantly lower than those of mice treated with vehicle control (Fig. 1B). And tumor growth was significantly suppressed by RocA (Fig. 1C) compared to vehicle control. Furthermore, flow cytometry analysis revealed that RocA significantly increased the tumor-infiltrating lymphocytes (TILs) (Fig. 1D and 1E), including both CD4^+^ and CD8^+^ T cells (Fig. 1D and 1F). The percentage of splenic CD4^+^ and CD8^+^ T cells was also increased by RocA treatment (Fig. 1G and 1H). These results demonstrated that RocA could promote T cells infiltrating to the tumor sites.

Tumors can be divided into immune-hot, -cold and -desert tumors according to the level of TILs. We further investigated the promoting effect of RocA on intra-tumoral T cells in B16F10 melanoma, a typical immune-cold tumor. RocA significantly decreased tumor weight and suppressed tumor growth of B16F10 melanoma (Fig.S1A, B). Similarly, RocA remarkably increased the percentage of tumor-infiltrating CD4^+^ and CD8^+^ T cells (Fig.S1C). Unexpectedly, RocA did not increase the percentage of splenic lymphocytes in tumor-bearing mice (Fig.S1D), which was not consistent with the results in Lewis lung cancer. Those results showed that RocA increased tumor-infiltrating T cells independent of the increasing of splenic T cells. Moreover, RocA inhibited the growth of immune-hot CT26 colon cancer in vivo, but did not affect the intratumoral infiltration of T cells (Fig. S2A-D), which could be attributed to the abundance of lymphocytes within immune-hot tumors.

Previously, we reported that RocA could promote the tumor infiltration of NK cells [25]. We next confirmed whether RocA-mediated intratumoral infiltration of T cells were dependent on NK cells. LLC tumor-bearing mice were treated with PK136 antibody to deplete NK cells in vivo (Fig.S3A, B). We found that NK cell depletion failed to abolish the RocA-mediated intratumoral infiltration of T cells (Fig.S3C). Similar results were observed in splenic T cells of tumor-bearing mice, RocA increased the proportion of T cells in the absence of NK cells (Fig.S3D). These results showed that RocA-mediated increase of TILs was independent of NK cells.

### The combination therapy of RocA and anti-PD-1 antibody exhibited potent antitumor activity in checkpoint-resistant tumor models

**Fig. 2:**
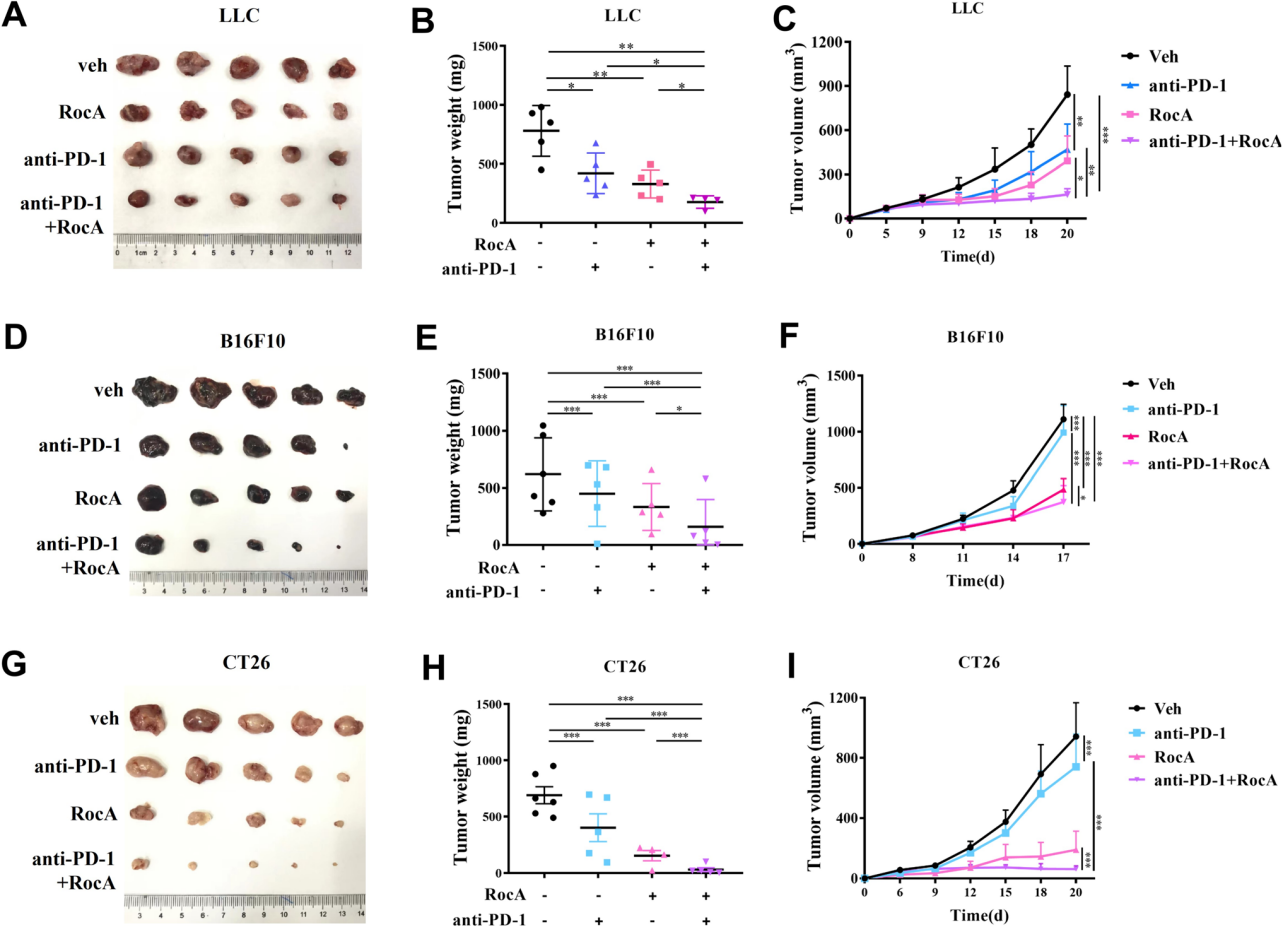
RocA promoted anti-tumor effect of anti-PD-1 antibody in checkpoint-resistant tumors. **A**–**C** LLC tumor-bearing C57BL/6 mice were administered with RocA every 2 days from day 3. And anti-PD-1 antibody was administered every 3 days from day 7. Tumor volume was measured at the indicated days. Mice were sacrificed at day 20 and tumors were excised, imaged and weighted. **D**–**F** B16F10 tumor-bearing C57BL/6 mice were administered with RocA every 2 days from day 3. And anti-PD-1 antibody was administered every 3 days from day 9. Tumor volume was measured at the indicated days. Mice were sacrificed at day 17 and tumors were excised, imaged and weighted. **G**–**I** CT26 tumor-bearing BALB/C mice were administered with RocA every 2 days from day 3. And anti-PD-1 antibody was administered every 3 days from day 10. Tumor volume was measured at the indicated days. Mice were sacrificed at day 20 and tumors were excised, imaged and weighted. *p < 0.05; **p < 0.01; ***p < 0.001

Promoting T cell infiltration to turn nonresponsive cold tumors into immune-responsive hot ones has become a breakthrough in enhancing the ICB therapy. The above-mentioned results demonstrated that RocA could transform immune-cold tumors into hot ones by fueling intratumoral infiltration of T cells. We next explored whether RocA can promote antitumor activity of anti-PD-1 antibody. We found that despite monotherapy of PD-1 blockade or RocA treatment suppressed tumor growth in a certain extent, the combination therapy of RocA and anti-PD-1 antibody exhibited the most potent suppressive effect on tumor growth (Fig. 2A-C). Moreover, we found that B16F10 melanoma cells and CT26 colorectal cancer cells were more resistant to checkpoint blockade therapy. The monotherapy of anti-PD-1 antibody showed minimal antitumor activity, while the combination therapy of RocA and anti-PD-1 antibody significantly suppressed tumor growth in both melanoma and colorectal cancer mouse models (Fig. 2D-I).

### The combination therapy of RocA and anti-PD-1 antibody overcomes checkpoint-resistant tumor via coordinated operations

**Fig. 3:**
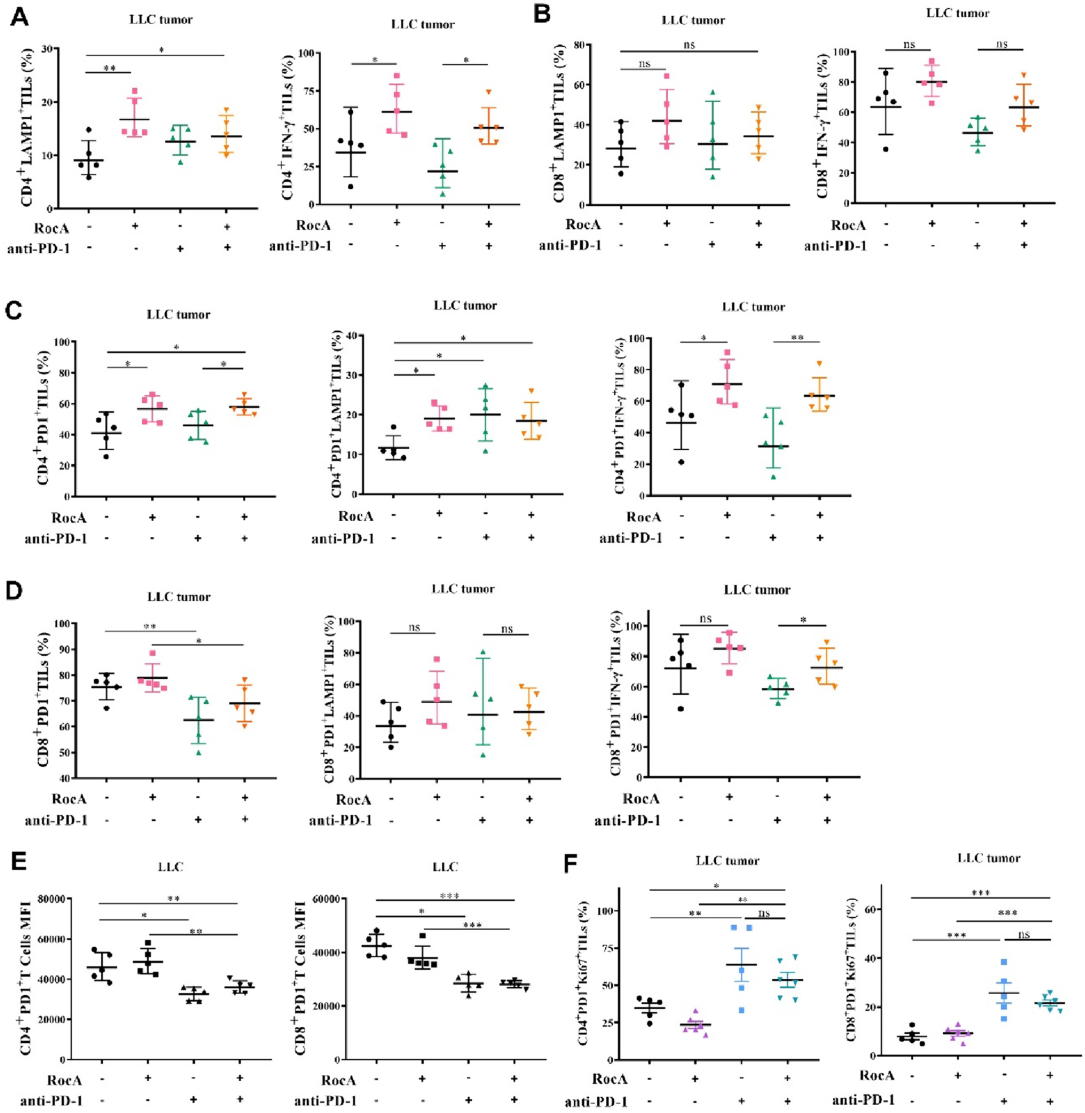
The combination of RocA and anti-PD-1 antibody promoted cytotoxicity and proliferation of TILs in LLC tumor model. A total of 1.5 × 10^6^ of LLC cells per mouse were subcutaneously inoculated on the upper back in C57BL/6 mice on day 0, and then 1.0 mg/kg of RocA was administered via i.p. injection every 2 days from day 3, anti-PD-1 antibody was administered via i.p. injection every 3 days from day 9. Tumor size was measured every 2 days, mice were sacrificed on day 19. Tumors were excised and underwent analysis through flow cytometry. **A** The expression of LAMP1 and intracellular production of IFN-γ in CD4^+^ TILs. **B** The expression of LAMP1 and intracellular production of IFN-γ in CD8^+^ TILs. **C** The expression of PD-1 on the surface of CD4^+^ TILs, and the expression of LAMP1 and intracellular production of IFN-γ in CD4^+^PD1^+^TILs. **D** The expression of PD-1 on the surface of CD8^+^TILs, and the expression of LAMP1 and intracellular production of IFN-γ in CD8^+^PD1^+^TILs. **E** The expression level of PD-1 receptor on the surface of PD1^+^ T cells. **F** The expression level of ki67 of PD1^+^ T cells. *p < 0.05; **p < 0.01; ***p < 0.001; ns, nonstatistical significance

We further explored the mechanism of action by which combination therapy suppressed tumor growth remarkably. As RocA increased tumor-infiltrating CD4^+^ and CD8^+^ T cells (Fig. 1D-F), we next investigated whether these TILs were functional in tumor microenvironment. Tumor tissues were collected from LLC tumor-bearing mice and tumor-infiltrating T cells were analyzed by flow cytometry. Indeed, RocA was found to significantly promote the degranulation and intracellular production of IFN-γ in CD4^+^ TILs but did not activate CD8^+^ TILs in LLC tumor tissues (Fig. 3A, 3B and 3D).

T cell activation is often associated with upregulation of PD-1. As expected, monotherapy of RocA significantly increased CD4^+^/PD-1^+^ T cells in LLC tumor tissues (Fig. 3C). We investigated whether anti-PD-1 antibody could prevent CD4^+^/PD-1^+^ TILs from checkpoint-mediated dysfunction. The results showed that CD4^+^/PD-1^+^ TILs were not downregulated in combination therapy group compared to those in RocA monotherapy group (Fig. 3C). However, the mean fluorescent intensity of PD-1 was significantly lower in combination therapy group compared to those in RocA monotherapy group (Fig. 3E). Moreover, the percentage of CD4^+^/PD-1^+^/Ki67^+^ and CD8^+^/PD-1^+^/Ki67^+^ cells was significantly increased by combination therapy compared to RocA monotherapy, which suggested that anti-PD-1 antibody promoted proliferation of TILs and helped RocA to overcome checkpoint-mediated inhibition of TILs (Fig. 3F). Taken together, these results suggested that RocA and anti-PD-1 antibody coordinated to promote immune response against checkpoint-resistant tumor.

### RocA upregulated genes involved in T helper cells

**Fig. 4:**
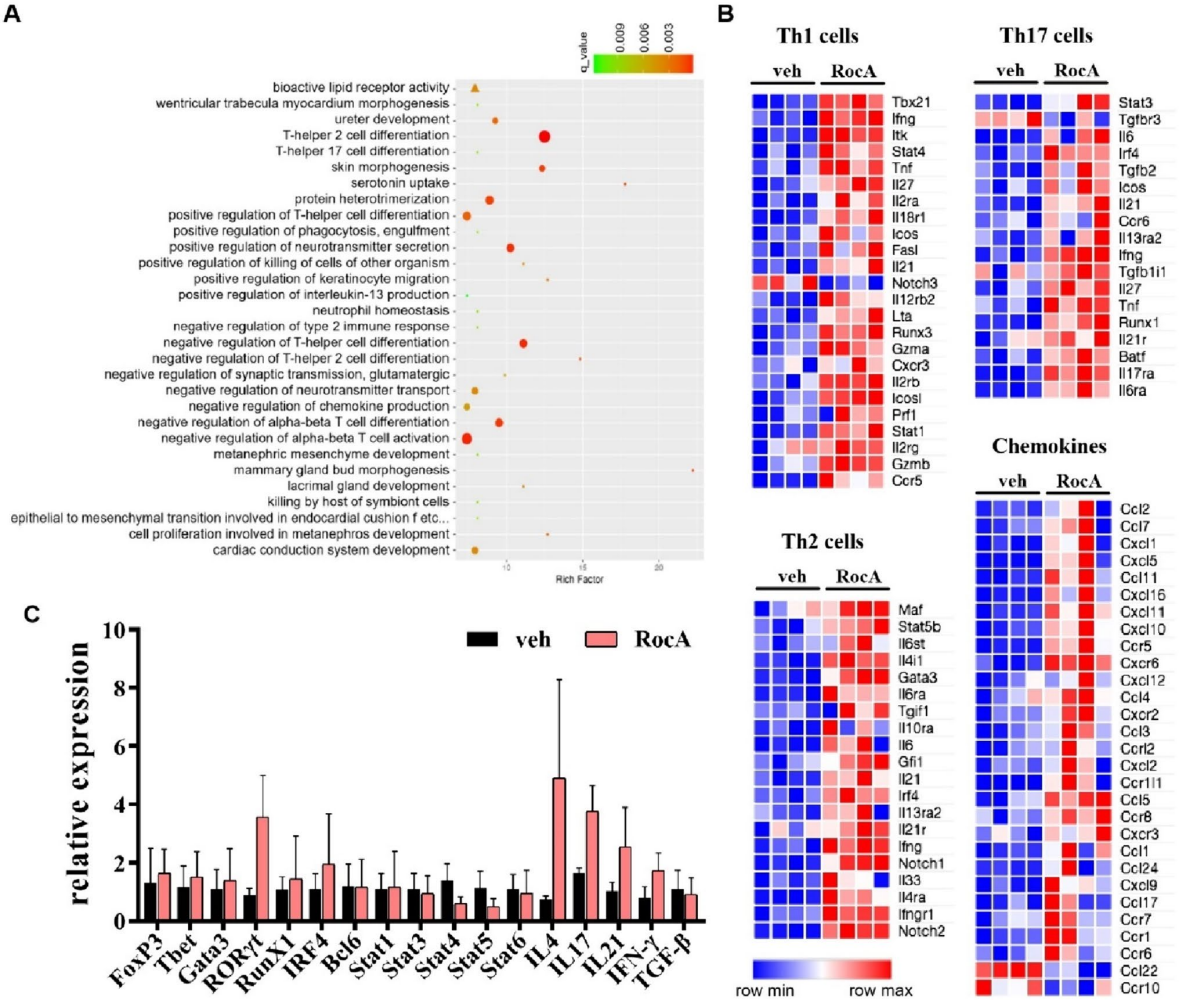
RocA regulates genes involved in T helper cells. A total of 1.5 × 10^6^ of LLC cells per mouse were subcutaneously inoculated on the upper back in C57BL/6 mice on day 0, and then 1.0 mg/kg of RocA was administered via i.p. injection every 2 days from day 3. Mice were sacrificed on day 17. Tumors were excised and underwent RNA sequencing. **A** The functional enrichment analysis of differential genes. **B** The gene expression of transcription factors, cytokines, effect factors and chemokines associated with Th1, Th2 and Th17 cells. **C** Validation of mRNA expression level of genes related to Th1, Th2 and Th17 cells via qRT-PCR

To explore the effects that RocA imposed on the tumor microenvironment, the tumor tissues were isolated and underwent analysis through RNA Sequencing. Strikingly, the functional enrichment analysis of differential genes showed that 5 of top 30 enrichments were about T helper cell differentiation (Fig. 4A). Therefore, we further analyzed the gene expression of transcription factors, cytokines, effect factors and chemokines associated with Th1, Th2 and Th17 cells, the three most common subtypes of T helper cells. We found that RocA did have a significant effect on the expression of related genes (Fig. 4B). The results of RNA-Seq were validated through qRT-PCR (Fig. 4C). These results indicated that RocA had an ability to regulate Th cell differentiation and effector function.

### RocA promoted CD4^+^ T cell differentiate into Th17 cells both in vivo and in vitro

**Fig. 5:**
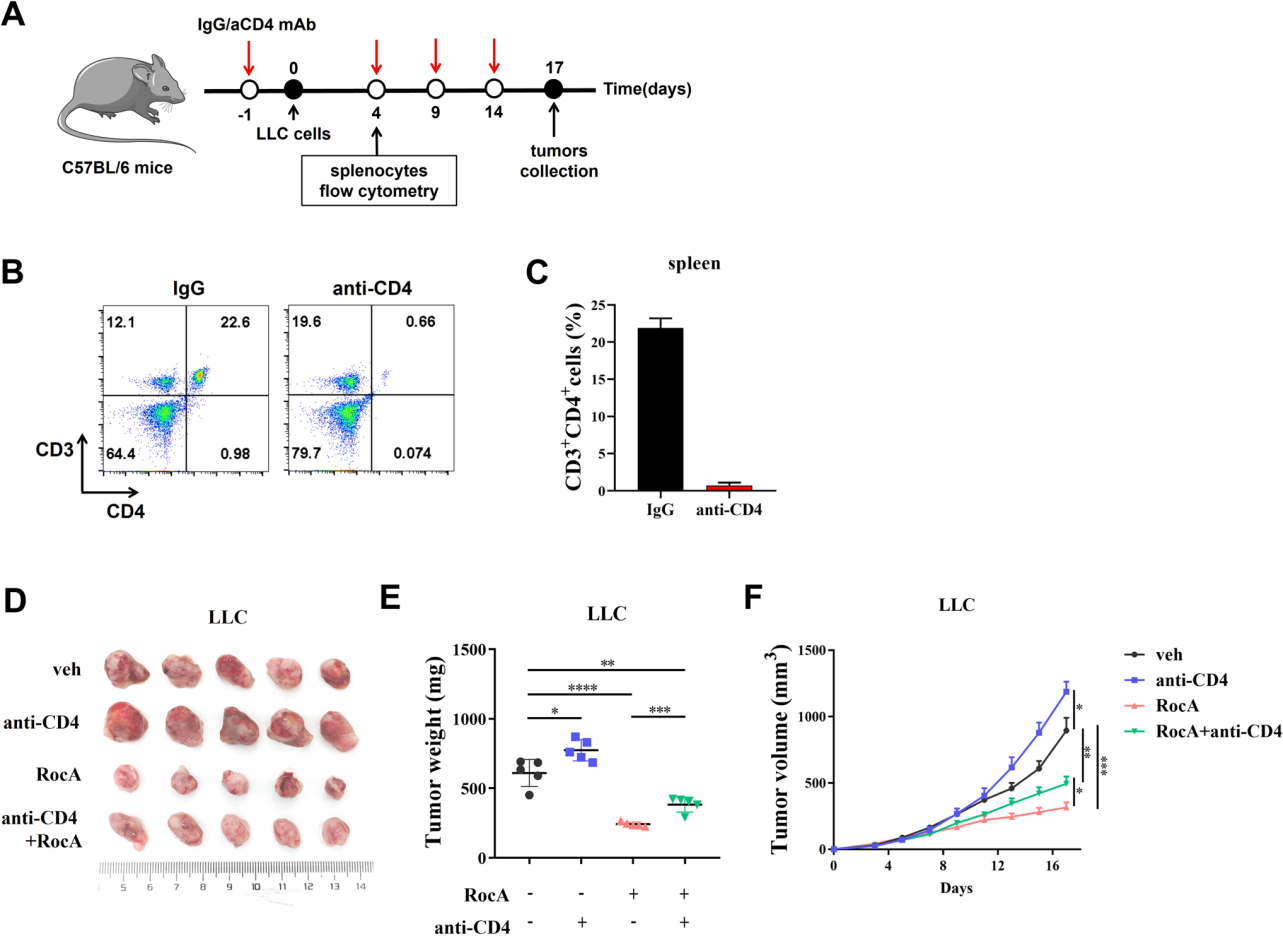
RocA promoted Th17 differentiation of CD4^+^ T cells. **A** 1.5 × 10^6^ LLC cells were subcutaneously injected into the right flank of C57BL/6 mice on day 0 and then 1.0 mg/kg of RocA was administered via i.p. injection every 2 days from day 3. Purified anti-mouse CD4 antibody was intraperitoneally injected every 5 days for CD4^+^ T cells depletion. **B** and **C** The splenocytes were isolated and used to detect the populations of CD3^+^CD4^+^ T cells through flow cytometry. **D**–**F** Tumor size was measured every 2 days, mice were sacrificed and tumors were excised, photographed and weighed. *p < 0.05; **p < 0.01; ***p < 0.001

We next investigated whether the antitumor activity of RocA is dependent on CD4^+^ T helper cells. Purified anti-mouse CD4 antibody was intraperitoneally injected for CD4^+^ T cell depletion (Fig. 5A), flow cytometry analysis showed that the CD4^+^ T cells of C57BL/6 mice were depleted by anti-CD4 antibody (Fig. 5B and C). We found that the antitumor activity of RocA was partially abolished by CD4^+^ T cell depletion (Fig. 5D-F), which indicated that CD4^+^ T cells play a pivotal role in mediating the antitumor activity of RocA.

**Fig. 6:**
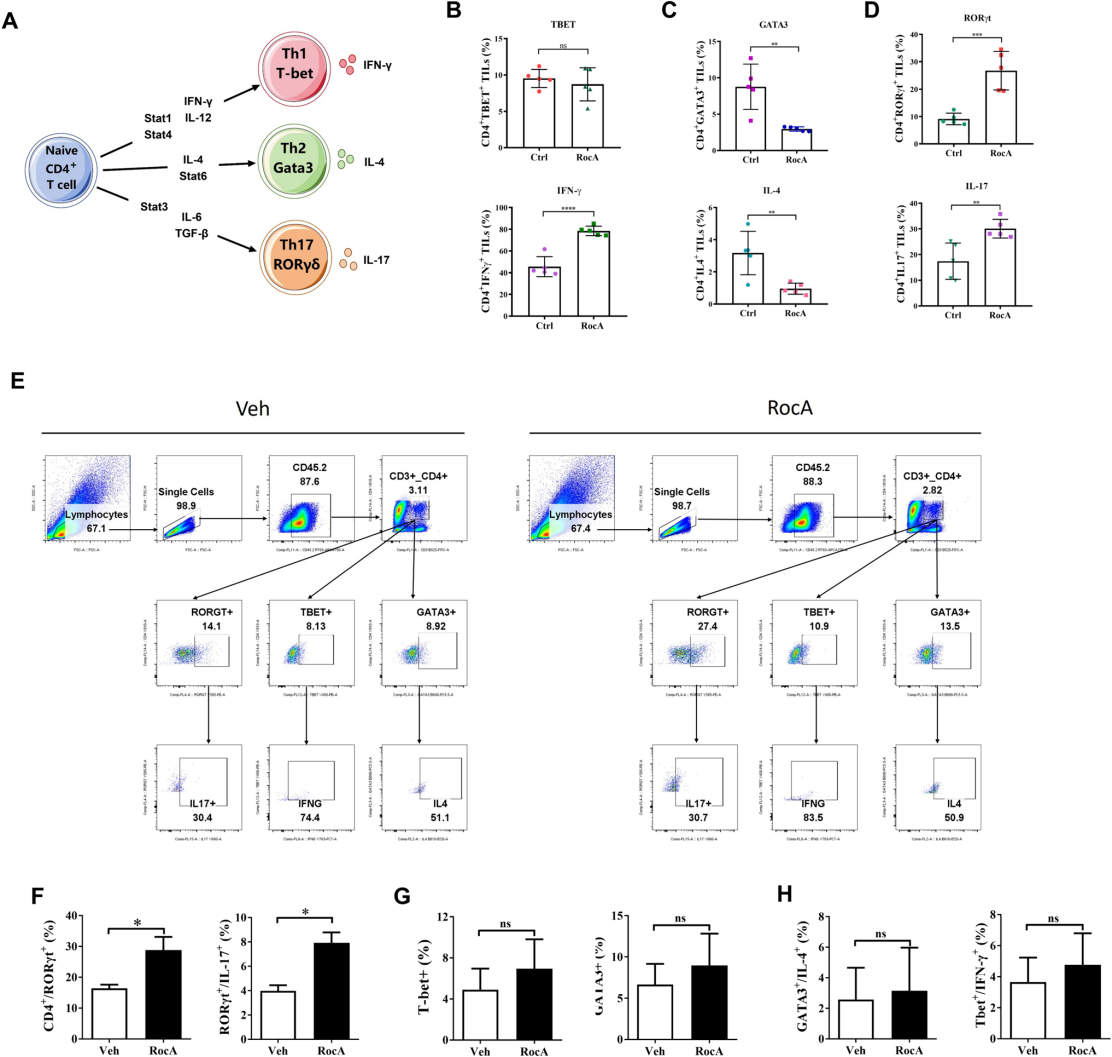
RocA promoted CD4^+^ TILs differentiate into Th17 cells. **A** The transcription factors and cytokines related to Th1, Th2 and Th17 cells. **B**, **C** A total of 1.5 × 10^6^ of LLC cells per mouse were subcutaneously inoculated on the upper back in C57BL/6 mice on day 0, and then 1.0 mg/kg of RocA was administered via i.p. injection every 2 days from day 3. Mice were sacrificed on day 17. Tumors were excised and underwent analysis through flow cytometry. **B **The proportion of CD4^+^TBET^+^TILs and CD4^+^IFNγ^+^TILs. **C** The proportion of CD4^+^GATA3^+^ TILs and CD4^+^IL4^+^TILs. **D** The proportion of CD4^+^RORγt^+^ TILs and CD4^+^IL17^+^TILs. **E**–**H** The splenic lymphocytes were isolate from healthy C57BL/6 mice and were then cultured in vitro and treated by IL-6, TGF-β and anti-CD3/CD28 beads. Intracellular staining of transcription factors and cytokines were conducted to analyze the CD4^+^ T cell subpopulation. *p < 0.05; **p < 0.01; ***p < 0.001; ns, nonstatistical significance

The CD4^+^ T cells mainly consist of T helper cells that can differentiate into Th1, Th2 and Th17 subpopulations (Fig. 6A). As the results of bulk RNA sequencing suggested that RocA might promoted Th17 differentiation of TILs. We sought to validate this concept both in vivo and in vitro. We investigated the CD4^+^ T cell subsets in RocA-treated tissues from tumor-bearing mice via intracellular stain flow cytometry. And the results showed that CD4^+^/RORγt^+^ population and CD4^+^/IL-17^+^ population, which mainly represents Th17 cells, were significantly increased after RocA treatment, while Th1 and Th2 cells were not increased by RocA (Fig. 6B-D). Moreover, splenic lymphocytes were isolate from healthy C57BL/6 mice and were then cultured in vitro. The cultured lymphocytes were treated with IL-6, TGF-β and anti-CD3/CD28 beads. Intracellular staining of transcription factors and cytokines were conducted to analyze the CD4^+^ T cell subpopulation (Fig. 6E-H). The results showed that RocA significantly increased the percentage of CD4^+^/RORγt^+^/IL-17^+^ subset, despite the percentage of IL-17^+^ cells in parental gate were not altered by RocA, which might be attributed to the release of IL-17. These results demonstrated that RocA promoted CD4^+^ T cell differentiate into Th17 cells.

## Discussion

The ICB therapy holds great promise as a revolutionary therapeutic approach, whereas the unsatisfactory response rates limited its application, which is mainly attributed to T cell dysfunction. In this study, we found that RocA transformed the immune-cold tumors into immune-hot ones through increasing the quantity and immunity of tumor-infiltrating T cells. On the other hand, the combination of RocA and anti-PD-1 antibody significantly downregulated the PD-1 expression and promote proliferation of TILs compared with those of RocA monotherapy. Moreover, we demonstrated that the antitumor activity of RocA depended in part on CD4^+^ T cells and RocA promoted the CD4^+^T cell differentiate into Th17 cells. These results revealed that RocA could be potentially applied in T cell-based cancer immunotherapy, especially in ICB therapy.

Immune checkpoint molecules regulate the immune system by stimulating and suppressing the immune response under physiological conditions, thus preventing excessive immune responses. PD-1 is expressed on the surface of activated T cells as an immune checkpoint molecule that negatively regulates T cell function to prevent inordinate killing [27]. PD-1 overexpression was initially thought to be a sign of T cell exhaustion [5]; however, recent studies have shown that PD-1 overexpression is actually a hallmark of high-functioning T cells [4]. In this study, we found that RocA upregulated the expression of PD-1, but we still observed increased effector function of these PD-1^+^T cells by detecting activation markers IFNγ and LAMP1, suggesting that RocA probably promoted the early killing of T cells, leading to the upregulation of PD-1 expression.

The interaction of PD-1 with PD-L1 and PD-L2 expressed by tumor cells has been suggested as a major mechanism of tumor immune evasion and is therefore an attractive target for cancer therapy [28]. Anti-PD-1 antibodies have been widely used in a variety of cancer types, including non-small-cell lung carcinoma, melanoma, colorectal cancer and renal cell carcinoma, and have shown significant antitumor activity over the past decade [29–32]. Nevertheless, some patients have demonstrated a lack of initial response to treatment (primary resistance) or patients with initial promising response to treatment can develop resistance overtime (acquired resistance), which necessitates improved treatment strategies [33]. Recently, many researches have been focused on the combination of anti-PD-1 antibody and other anti-tumor therapies such as anti-CTLA-4 agents, chemotherapy, radiotherapy and so on [34–36]. In this study, we demonstrated that the combination of RocA and anti-PD-1 antibody exerted potent antitumor activity even on the immune-cold B16F10 melanoma, a type of tumor with poor response to ICB therapy.

Indeed, RocA alone increased the quantity of intratumoral T cells while also upregulating tumor PD-L1 expression (data not shown), which to some extent increased the likelihood of T cell exhaustion. Although anti-PD-1 antibody alone can block the T cell inhibitory signaling, intratumoral infiltrating T cells remains insufficient to maximize the anti-tumor immune response, which is one of the major challenges facing ICB therapy. However, the combination of RocA and anti-PD-1 antibody increased intratumoral T cells and prevented T cell dysfunction, ultimately achieving a robust anti-tumor immune response.

Moreover, in the context of immune-hot CT26 colon cancer, RocA demonstrated a definite anti-tumor effect and promoted tumor suppressive activity of anti-PD-1 antibody, but RocA did not increase the quantity of T cells in the spleen and tumor microenvironment of mice. This discrepancy could be attributed to the abundance of lymphocytes within immune-hot tumors. Thus, lymphocyte function emerges as a critical factor influencing the anti-tumor effect of RocA, warranting further investigation.

Th17 cells, a subset of T helper cells, are originally identified and studied in detail in autoimmune diseases and have been associated with a variety of inflammatory contexts [37, 38]. The differentiation of Th17 cells is definitively orchestrated by the transcription factor orphan nuclear receptor RORgammat (RORγt) [39]. Th17 cells express the cytokines IL-17, IL-21, IL-22 and other cytokines such as IFNγ and TNFα in human tumor tissues [40]. The debate about whether tumor-infiltrating Th17 cells inhibit or promote cancer progression continues, the nature of these cells depends on the various cancer environment [41–43]. In fact, Th17 cells can be further categorized into various lineages based on the specificity of cytokines production. In this study, we observed that RocA promoted CD4^+^ T cells to differentiate into Th17 cells. However, the specific role of RocA in promoting tumor-infiltrating Th17 cells and the underlying mechanism still require further elucidation.

In summary, our study elucidated that RocA could facilitate T cell anti-tumor immunity and combine with anti-PD-1 antibody against tumors. Consequently, RocA holds promise for enhancing the efficacy of cancer immunotherapy, offering potential solutions to challenges posed by existing immunotherapies such as immune checkpoint blockade therapy.

## Conclusion

This study reported the therapeutic potential of combination therapy of RocA and anti-PD-1 antibody. We found that RocA promoted the infiltration and differentiation of CD4^+^ TILs and coordinated with anti-PD-1 antibody to overcome checkpoint resistance in multiple tumor models. Indeed, RocA showed a capacity of fueling the T cell anti-tumor immunity and serving as a therapeutic candidate in enhancing the ICB therapy.

### Supplementary Information

Below is the link to the electronic supplementary material.

Supplementary file1 (JPG 202 KB)

Supplementary file2 (JPG 191 KB)

Supplementary file3 (JPG 216 KB)

## Data Availability

The data are available from the corresponding author on reasonable request.

## Abbreviations

CD: Clusters of differentiation
cGAS: Cyclic GMP-AMP synthase
FOXP3: Forkhead box protein P3
GATA3: GATA binding protein 3
ICB: Immune checkpoint blockade
IFN-γ: Interferon gamma
IL: Interleukin
LAMP1: Lysosomal-associated membrane protein 1
NK: Natural killer cell
PD-1: Programmed cell death protein 1
PD-L1: Programmed death-ligand 1
PD-L2: Programmed death-ligand 2
RocA: Rocaglamide
RORγt: RAR-related orphan receptor gamma
RNA-Seq: RNA sequencing
STING: Stimulator of interferon genes
TBET(TBX21): T-box transcription factor 21
Tfh: Follicular helper T cell
TGF-β: Transforming growth factor beta
Th: T helper cell
TIL: Tumor-infiltrating lymphocyte
Treg: Regulatory T cell
ULK1: Unc-51-like kinase 1
