# Supplementary figures and images for: Rocaglamide promotes infiltration and differentiation of T cells and coordinates with PD-1 inhibitor to overcome checkpoint resistance in multiple tumor models
Source: Cancer Immunol Immunother. 2024 Jun 4;73(8):137. doi: 10.1007/s00262-024-03706-5 (PMC11150362; doi:10.1007/s00262-024-03706-5)

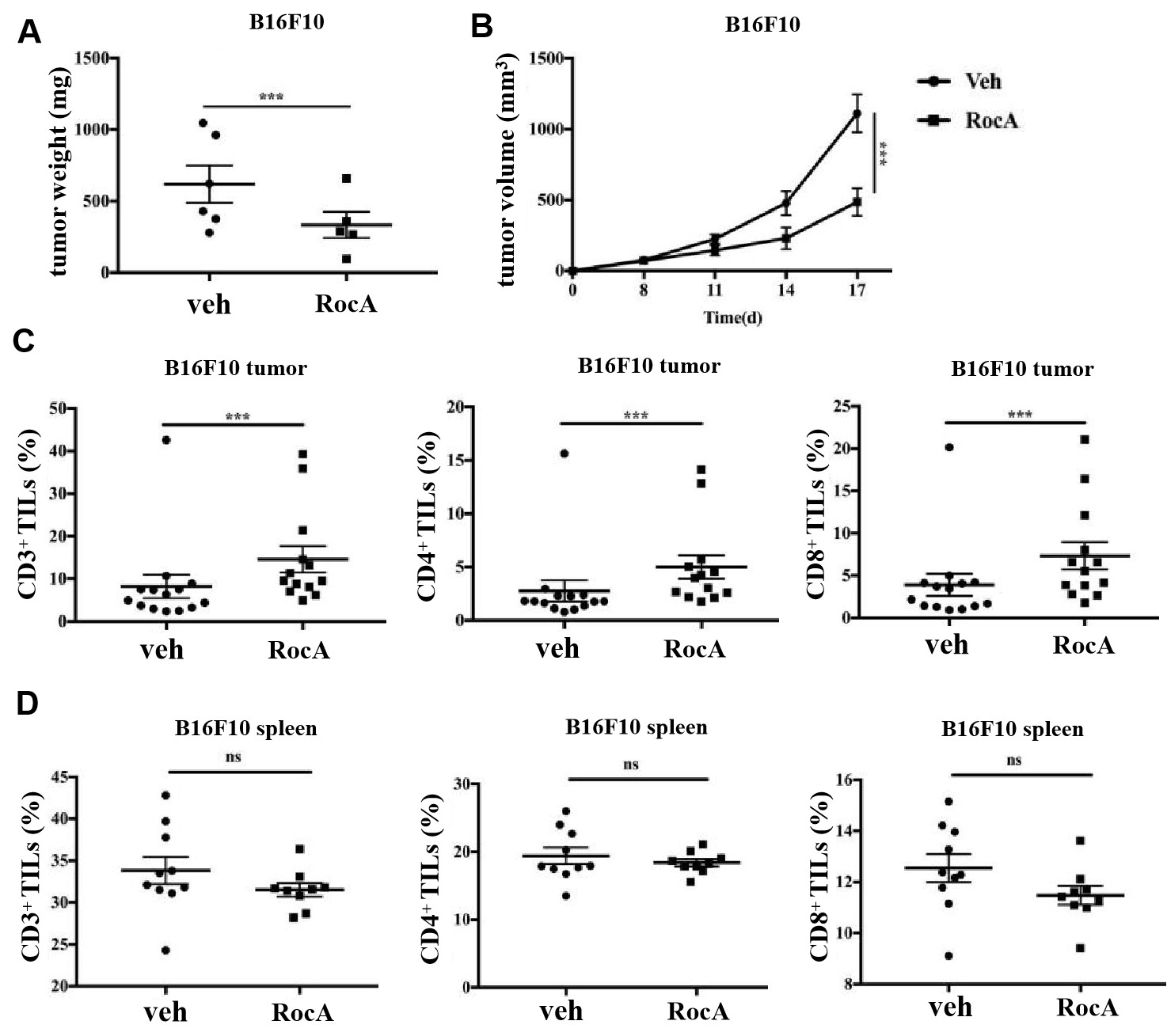

Supplement: Supplementary file 1 — Supplementary file1 (JPG 202 KB) [file 262_2024_3706_MOESM1_ESM.jpg]

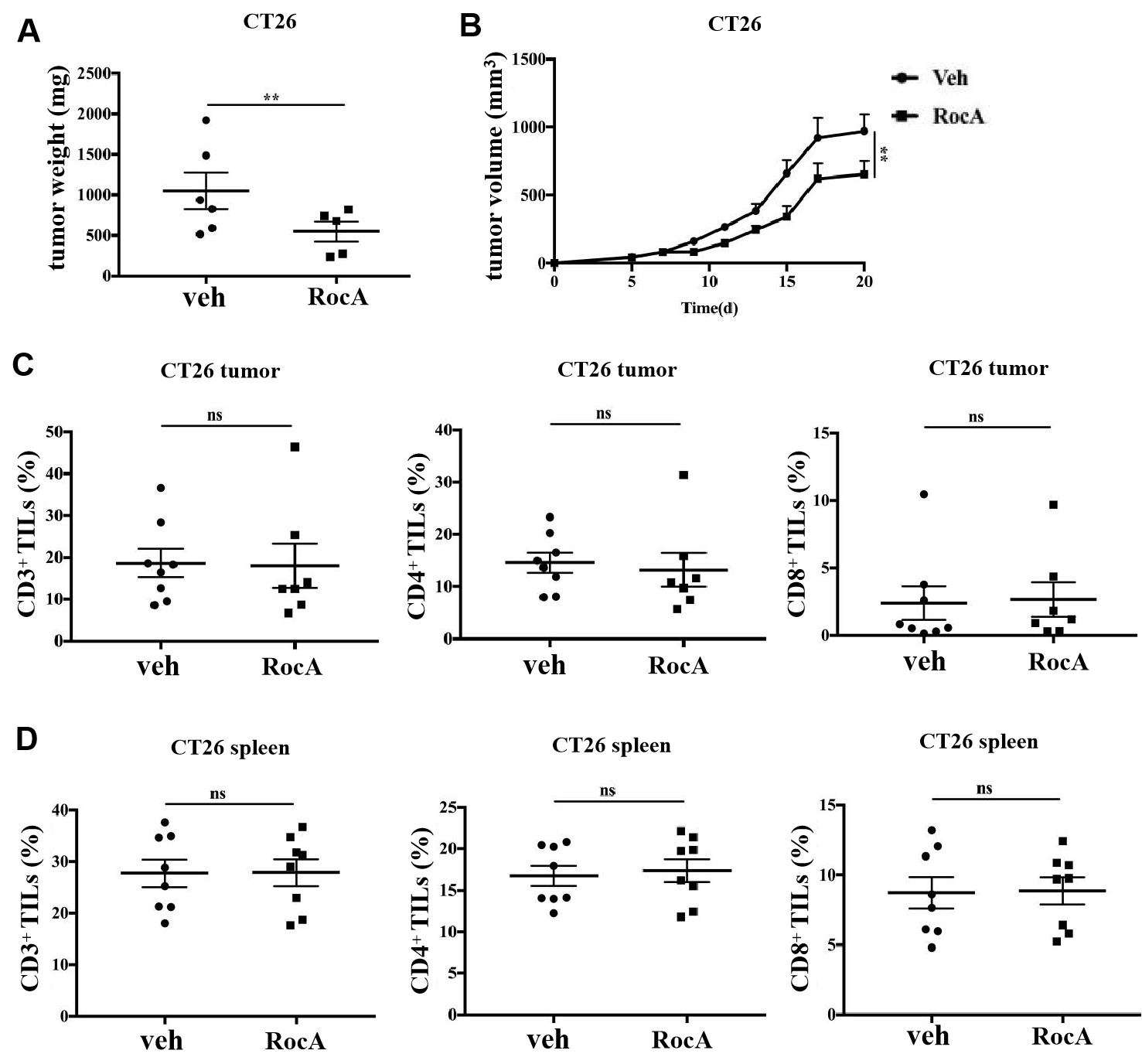

Supplement: Supplementary file 2 — Supplementary file2 (JPG 191 KB) [file 262_2024_3706_MOESM2_ESM.jpg]

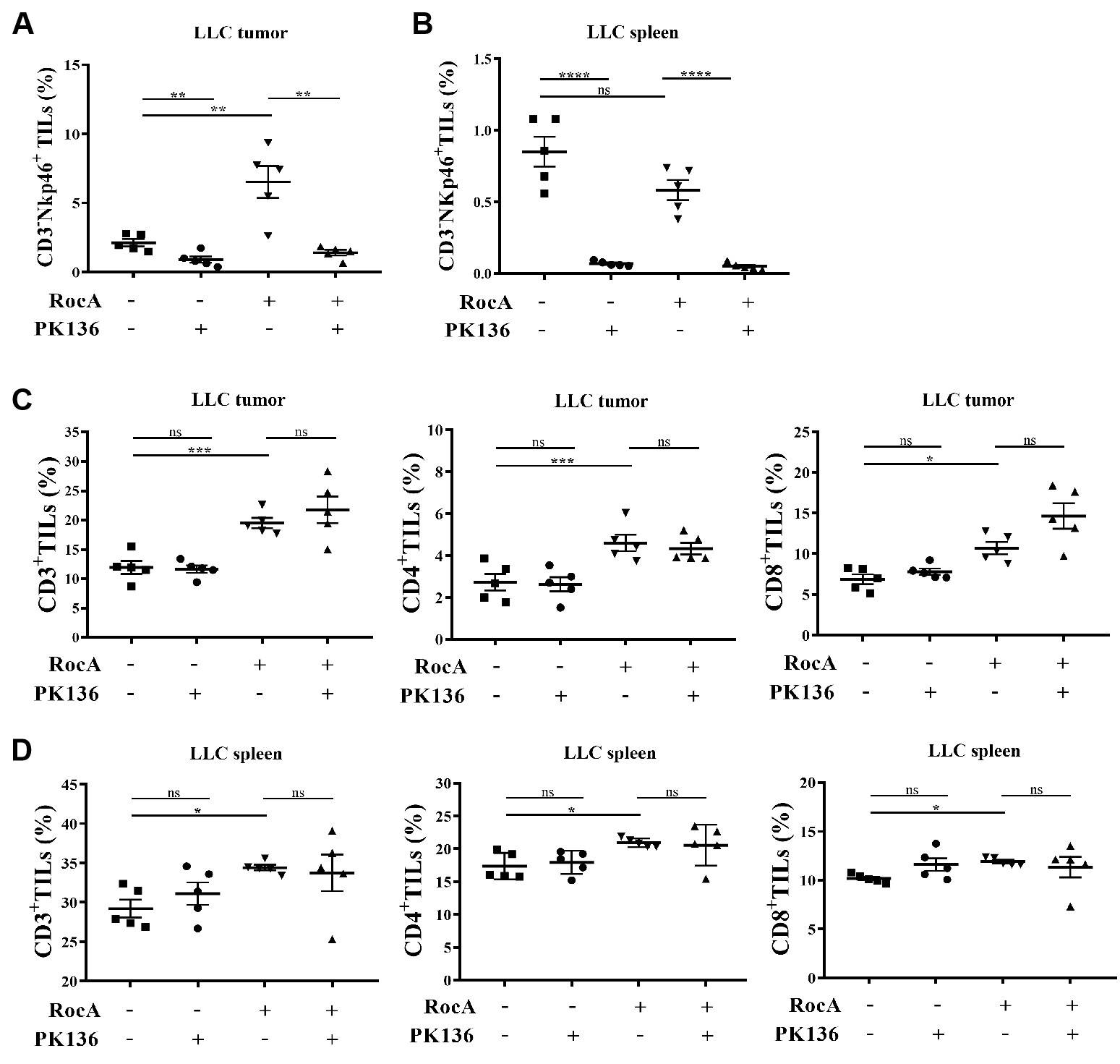

Supplement: Supplementary file 3 — Supplementary file3 (JPG 216 KB) [file 262_2024_3706_MOESM3_ESM.jpg]
